# Development of PancRISK, a urine biomarker-based risk score for stratified screening of pancreatic cancer patients

**DOI:** 10.1038/s41416-019-0694-0

**Published:** 2019-12-20

**Authors:** Oleg Blyuss, Alexey Zaikin, Valeriia Cherepanova, Daniel Munblit, Elena M. Kiseleva, Olga M. Prytomanova, Stephen W. Duffy, Tatjana Crnogorac-Jurcevic

**Affiliations:** 1 Centre for Cancer Prevention, Wolfson Institute of Preventive Medicine, Queen Mary University of London, London, UK; 2 School of Physics, Astronomy and Mathematics, University of Hertfordshire, Hatfield, UK; 3 Department of Paediatrics and Paediatric Infectious Diseases, Institute of Child Health, Sechenov First Moscow State Medical University, Moscow, Russia; 4 Department of Mathematics and Institute for Women’s Health, University College London, London, UK; 5 Department of Applied Mathematics, Lobachevsky State University of Nizhny Novgorod, Nizhny Novgorod, Russia; 6 Inflammation, Repair and Development Section, National Heart & Lung Institute, Imperial College London, London, UK; 7 Oleh Honchar Dnipro National University, Dnipro, Ukraine; 8 Centre for Molecular Oncology, Barts Cancer Institute, Queen Mary University of London, London, UK

**Keywords:** Tumour biomarkers, Cancer screening

## Abstract

**Background:**

An accurate and simple risk prediction model that would facilitate earlier detection of pancreatic adenocarcinoma (PDAC) is not available at present. In this study, we compare different algorithms of risk prediction in order to select the best one for constructing a biomarker-based risk score, PancRISK.

**Methods:**

Three hundred and seventy-nine patients with available measurements of three urine biomarkers, (LYVE1, REG1B and TFF1) using retrospectively collected samples, as well as creatinine and age, were randomly split into training and validation sets, following stratification into cases (PDAC) and controls (healthy patients). Several machine learning algorithms were used, and their performance characteristics were compared. The latter included AUC (area under ROC curve) and sensitivity at clinically relevant specificity.

**Results:**

None of the algorithms significantly outperformed all others. A logistic regression model, the easiest to interpret, was incorporated into a PancRISK score and subsequently evaluated on the whole data set. The PancRISK performance could be even further improved when CA19-9, commonly used PDAC biomarker, is added to the model.

**Conclusion:**

PancRISK score enables easy interpretation of the biomarker panel data and is currently being tested to confirm that it can be used for stratification of patients at risk of developing pancreatic cancer completely non-invasively, using urine samples.

## Background

Since the Framingham study in 1976, yielding a first risk prediction model for coronary heart disease, a number of prediction models have been reported for various medical conditions, including cancer.^1–5^ In pancreatic ductal adenocarcinoma (PDAC), few such models have been designed, including the ones for absolute risk prediction^6–12^ and gene carrier status prediction,^13^ as well as prediction models in groups at risk.^14,15^ Recently, two independent models to determine the risk of PDAC in patients in new-onset diabetes (NOD) cohort have also been reported.^16,17^ Most of these prediction models are based on previously established risk factors, relevant laboratory findings and clinical symptoms, but none have as yet been thoroughly validated or adopted in the clinic.

We have recently reported on three-biomarker panel in urine with promising characteristics for early detection of PDAC.^18^ In order to enable its utilisation and allow for seamless result interpretation in the clinical setting, we aimed to develop a risk score based on these three biomarkers, age and urine creatinine. In order to ascertain whether the most appropriate and best performing model is utilised, we have compared several different algorithms: neural network (NN), random forest (RF), support vector machine (SVM), neuro-fuzzy (NF) technology, and logistic regression model. These are all supervised methods that require a training set of patients with known case/control labels. Following the training stage, all these methods could be applied to new patients, which would give the risk of the disease or the exact prognosis of the class (case/control) label.

Each of these methods has its advantages and disadvantages. The most widely used approach in clinical studies is multivariable regression, with logistic regression being the most appropriate for the binary outcome (case/control).^19^ It includes continuous, categorical and ordinal variables and does not require a normal distribution of the predictors while providing coefficients that can be easily converted into odds ratios (ORs) with straightforward interpretation. Another method, Deep Learning, has also been widely applied to different biomedical data sets.^20,21^ Although deep NNs are more suitable for large data sets, they have also been successfully utilised for a small volume medical data.^22^

RF is another common machine learning technique utilised for building the predictive models. It is an ensemble learning method for classification based on the Condorcet’s jury theorem stating that a set of competent, independent jurors that are making a decision on a binary outcome using the majority voting scheme will be more effective with the increasing number of jurors. One of the main advantages of this approach is that combining multiple decision trees avoids overfitting.^23–25^ Similarly, SVM is a supervised learning algorithm that transforms the original input space into a higher-dimensional feature space to find the hyperplane that separates the classes in an optimal way. The “penalty” term that controls the trade-off between margin and training errors prevents the overfitting of the model.^26^

A more recent technique, NF technology, models complex processes and solves the optimal set partitioning problems in case of uncertainty.^27,28^ This approach unites two independent mathematical constructions, fuzzy logic^29^ and NNs, which offers the possibility to combine the ability of NNs to learn with transparency and easy interpretation of fuzzy rules “If–Then”.^30,31^

All five algorithms were tested; they were first trained on a subset of data and subsequently validated using the remaining subset.

## Materials and methods

### Clinical sample set for the analysis

The data utilised for this analysis was obtained by enzyme-linked immunosorbent assays for the three biomarkers on the specimens collected at the Royal London Hospital, University College London Hospital, Department of Surgery, Liverpool University and the CNIO Madrid, Spain, combined with creatinine and patient’s age as described in ref. ^18^ In addition to the already available data, further samples obtained from Pancreas Tissue Bank (https://www.bartspancreastissuebank.org.uk) were also analysed in the same fashion, deriving a total set of 180 healthy controls and 199 PDAC samples (102 stage I/II and 97 stage III/IV) (these data will be reported in more detail separately). The analysis was performed with Ethical approval given by North East-York Research Ethics Committee (Ref: 18/NE/0070).

### Training of algorithms

Logistic regression, NN, RF, SVM and NF technology were trained in the training set and tested in the validation set after random division in a 1:1 ratio. The training set included both PDAC and healthy patients.

A logistic regression model was fitted for the training set using the five predictors—three urine biomarkers together with creatinine and age. Bootstrap cross-validation was used for the internal validation to ensure that the overfitting is avoided.^32^ Following that, elastic net was used for the regularisation of the coefficients to obtain the final model.^33^ The “glmnet” package from R was used to implement the logistic regression model with elastic net regularisation.

The depth and architecture of NNs was varied in our study. In particular, NNs with 1–16 hidden layers with increasing number of neurons from 16 neurons in the first layer to 256 neurons in the last layer were tried. Also, different optimisers, learning rates and activation functions were attempted. As a result, the optimal model was found empirically and consisted of 7 feed-forward hidden layers with 32, 32, 64, 64, 128, 128 and 2 neurons, respectively, and 6 dropout layers with probability equal to 0.2 in between the hidden layers. The NN was trained on standardised features. Finally, the NN was trained for 100 epochs with batch size of 16 using the Adam optimiser with learning rate of 0.001. To implement the model and test its performance, the following Python packages were used: tensorflow, keras, and scikit-learn.^34^

The RF of conditional inference trees was fitted on the training set. The “party” package from R was used and then applied to the validation set to test its performance. Such implementation provided fixed values for sensitivity and specificity in the validation set rather than a range of values, therefore the area under the Receiver Operating Characteristic (ROC) curve (AUC) was not calculated for this approach.

To select optimal parameters of SVM,^35^ a ten-fold cross validation was used. The “svmLinear” method from the “caret” package in R was used to train and test the SVM.

For tuning of the NF method, the r-algorithm developed by Shor was used with a precision *ε* *=* 0.001.^36^ Software implementation of this approach was developed within the Visual Studio 2013 environment.

### Statistical analysis

The outcome of the analysis was PDAC diagnosis.

The null hypothesis in this study was that the logistic regression model, the easiest to implement and evaluate from the list of algorithms, performs no worse than any of the more sophisticated techniques.

The performance characteristics of the algorithms were evaluated and compared in terms of the sensitivity (SN; proportion detected of those with cancer) at a fixed specificity (SP; proportion of healthy controls correctly detected not to have cancer); for RF and SVM, the threshold was implicit in its formulation; for logistic regression, NN and NF technology, the threshold was the value that provided an SP of 0.90; and the AUC. Inference for the ROC curves was based on cluster-robust standard errors that accounted for the serially correlated nature of the samples. It was not possible to create ROC curves and therefore AUC for RF and SVM since the outcome was not continuous. McNemar’s exact test was used to assess the significance of difference in SN at fixed SP and DeLong’s test was used to assess the significance of differences in AUC between approaches.^37^ Confidence intervals (CI 95%) for AUCs were derived based on the DeLong’s method to evaluate the uncertainty of an AUC; SN and SP 95% CI were derived using bootstrap replicates.

To allow for multiple testing, both types of tests were adjusted using the Bonferroni correction. Since the primary hypothesis pertained to the logistic regression model, all other approaches were compared to this model, and a threshold of 0.05/4 = 0.0125 was used to define a significant result after adjustment for multiplicity.

All analyses were performed in R version 3.5.1 and Python version 3.0.

## Results

**Fig. 1:**
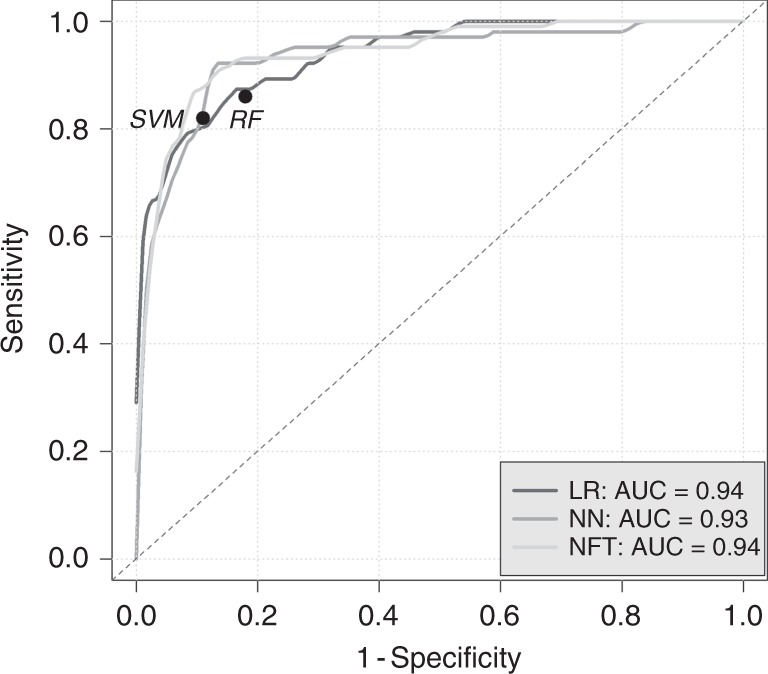
Performance characteristics of urine biomarkers interpreted using logistic regression, neural network, neuro-fuzzy technology, random forest and support vector machine for detection of pancreatic cancer (PDAC) cases. Circle points give particular values of sensitivity and specificity provided by random forest and support vector machine. LR logistic regression, NN neural network, NFT neuro-fuzzy technology, RF random forest, SVM support vector machine, AUC area under ROC curve.

In total, 379 samples were included in the analysis. The training and validation sets comprised of 191 patients (96 PDAC cases and 95 controls) and 188 patients (103 PDAC and 85 controls), respectively. Characteristics of samples were balanced (Table 1). Following the training stage, all the algorithms were applied to the validation set. Figure 1 shows the ROC curves for the logistic regression, NN and NF technology for detection of PDAC cases. Circle points on the ROC curves give particular values of SN and SP provided by SVM and RF. Logistic regression and NF technology provided the same AUC, 0.94 (95% CI: 0.91–0.97), slightly higher than the figure of 0.93 (95% CI: 0.9–0.97) for the NN; however, the difference was not significant (*p* = 0.26 for logistic regression vs NN and *p* = 0.24 for NF technology vs NN). At a fixed SP of 0.9, SN was 0.81 (95% CI: 0.7–0.89) for logistic regression, 0.81 (95% CI: 0.63–0.95) for NN and 0.87 (95% CI: 0.72–0.95) for NF technology (Table 2). Since the outcome of the SVM and RF algorithms was not continuous, these are included with actual specificities that they provided.

**Table 1:** Details of cases and controls in the training and validation sets.

		Age	Creatinine (g/L)	LYVE1 (ng/mL)	REG1B (ng/mL)	TFF1 (ng/mL)
Healthy samples	Training set (*n* = 95): mean (sd)	56.495 (12.095)	0.73 (0.543)	1.196 (1.994)	43.271 (72.339)	135.779 (224.582)
	Testing set (*n* = 85): mean (sd)	56.518 (12.121)	0.851 (0.574)	1.231 (1.871)	38.855 (49.385)	193.749 (325.936)
	*p* value	0.999	0.107	0.444	0.616	0.11
PDAC samples	Training set (*n* = 96): mean (sd)	66.271 (9.934)	0.94 (0.678)	5.542 (3.662)	216.731 (262.845)	1124.184 (1158.383)
	Testing set (*n* = 103): mean (sd)	66.097 (11.066)	0.894 (0.767)	6.019 (3.891)	235.235 (290.935)	1171.382 (1641.401)
	*p* value	0.979	0.244	0.395	0.674	0.773

**Table 2:** Cut-point sensitivity, specificity, area under curve (AUC).

Method	Logistic regression	Neural network	Neuro-fuzzy technology	SVM	Random forest
Specificity	0.9	0.9	0.9	0.89	0.82
Sensitivity (95% CI)	0.81 (0.7–0.89)	0.81 (0.63–0.95)	0.87 (0.72–0.95)	0.82	0.86
*p* value		1	0.077	1	0.18
AUC (95% CI)	0.94 (0.91–0.97)	0.93 (0.9–0.97)	0.94 (0.91–0.97)		

To assess the significance of differences in sensitivity at fixed specificity for different algorithms, McNemar’s exact test was used and adjusted for the multiple comparison of four algorithms with the logistic regression. As seen in Table 2, none of the approaches significantly outperformed logistic regression implying that the null hypothesis cannot be rejected. In a subgroup analysis of early and late PDAC stage (Table 3), performance was similar with differences in AUC between the logistic regression and other techniques being negligible. Therefore, logistic regression was implemented into a PancRISK using all the available data.

**Table 3:** Area under curve (AUC) in early and late PDAC stage subgroups.

Method	Logistic regression	Neural network	Neuro-fuzzy technology
Healthy (*n* = 85) vs Stage I, II PDAC (*n* = 56) AUC (95% CI)	0.95 (0.92–0.98)	0.93 (0.89–0.98)	0.93 (0.88–0.97)
Healthy (*n* = 85) vs Stage III, IV PDAC (*n* = 47) AUC (95% CI)	0.93 (0.88–0.97)	0.93 (0.88–0.98)	0.96 (0.93–0.99)

To analyse whether CA19-9, a commonly used pancreatic cancer biomarker, is complementary to the developed PancRISK, both were evaluated in the subset of data where plasma CA19-9 measurements were available. Samples were classified by the PancRISK as “Normal” or “Abnormal” based on the threshold that provided the specificity of 0.9 while for CA19-9 the clinically used cut-off of 37 U/mL was used. Table 4 shows the number of healthy and PDAC samples that were classified as “Normal” and “Abnormal” using the PancRISK and CA19-9 37 U/mL cut-off. The rule of “Either PancRISK or CA19-9 is Abnormal” provided specificity of 87/91 = 0.96 and sensitivity of 144/150 = 0.96.

**Table 4:** Classification of the subset of healthy and PDAC samples using PancRISK and CA19-9 cut-off of 37 U/mL.

Healthy (*n* = 91)	CA19-9 < 37 U/mL	CA19-9 ≥ 37 U/mL	Specificity: 0.96
PancRISK = “Normal”	87	3	
PancRISK = “Abnormal”	1	0	
PDAC (*n* **=** 150)	CA19-9 < 37 U/mL	CA19-9 ≥ 37 U/mL	Sensitivity: 0.96
PancRISK = “Normal”	6	30	
PancRISK = “Abnormal”	15	99	

## Discussion

With increased incidence and no major improvements in detection and therapeutic approaches, PDAC stubbornly remains one of the few cancers with exceptionally poor prognosis. We believe that earlier cancer detection, when still in fully resectable stage, using a non-invasive testing will likely be critical in improving the currently bleak outcome for pancreatic cancer patients. Owing to fairly small incremental increase in overall risk even when several well-known risk factors are combined, with or without adding PDAC symptoms (due to their late occurrence and non-specific nature), prediction risk models based on molecular biomarkers are more likely to accelerate earlier detection of PDAC.

In this study, in order to assemble a biomarker-based risk score, we have used our urinary biomarker data to compare five different classification techniques: logistic regression, NN, RF, SVM, and NF technology, and found that all of them had performed similarly and therefore the null hypothesis about their equality cannot be rejected. Since the logistic regression was not outperformed by any of the more sophisticated approaches, it was implemented in the construction of PancRISK score. This choice is substantiated by the fact that, out of all the utilised algorithms, it is the most straightforward to implement and interpret.

The performance of PancRISK was subsequently compared toplasma CA19-9 in a subset of data where matched measurements were available. The comparison indicated that this combination could provide very high sensitivity and specificity of PDAC detection.

The intended use of PancRISK is in stratification of patients to the ones with normal (“Normal”) or elevated (“Abnormal”) risk, with further, more expensive and invasive clinical workup being indicated in the latter group. The PancRISK could thus be utilised in the surveillance of individuals with familial history and genetic background or in patients with increased risk due to inflammatory diseases of pancreas, such as chronic pancreatitis. Furthermore, it would also be interesting to assess the model in the PC-NOD group with intermediate ENDPAC score.^17^

Our study has several limitations, the main one being that, while we aim to detect cancer at an earliest possible stage, about half of PDAC cases in our data set were late-stage patients. This is due to challenges in finding PDAC patients with early-stage disease, as most are currently diagnosed when the disease is either locally advanced or already metastatic. Similarly, we have used healthy people as a proxy for individuals with genetic background until such samples become available to us. Additional limitation concerns the analysis of PancRISK in combination with CA19-9, where both measurements were available only in a subset of patients. The main strength of our study, however, is the comprehensive comparison of five different classification algorithms, which was our main goal. As there are only five predictors used in building our predictive models, the ten events per variable rule of thumb is easily satisfied.^38^ Thus the volume of data analysed here enabled us to conclude that the logistic regression is the appropriate model for building the prediction of PDAC risk.

The performance of PancRISK now requires further evaluation in the large number of prospectively collected specimens in a setting of a clinical observational study, both alone and in the combination with CA19-9, which will give a definitive estimate of the predictive power of such a combination.

## Data Availability

The data that support the findings of this study are available on request from the corresponding author.

## References

[1] Cassidy A, Duffy SW, Myles JP, Liloglou T, Field YK (2006). Lung cancer risk prediction: a tool for early detection. Int. J. Cancer.

[2] Wang X, Oldani MJ, Zhao X, Huang X, Qian Q (2014). A review of cancer risk prediction models with genetic variants. Cancer Inform..

[3] Tyrer J, Duffy SW, Cuzick J (2004). A breast cancer prediction model incorporating familial and personal risk factors. Stat. Med..

[4] Wen CP, Lin J, Yang YC, Tsai MK, Tsao CK, Etzel C (2012). Hepatocellular carcinoma risk prediction model for the general population: the predictive power of transaminases. J. Natl Cancer Inst..

[5] Blyuss O, Burnell M, Ryan A, Gentry-Maharaj A, Marino I, Kalsi J (2018). Comparison of longitudinal algorithms as first line tests for ovarian cancer screening: a nested cohort study within UK Collaborative Trial of Ovarian Cancer Screening (UKCTOCS). Clin. Cancer Res..

[6] Zhao D, Weng C (2011). Combining PubMed knowledge and HER data to develop a weighted Bayesian network for pancreatic cancer risk prediction. J. Biomed. Inform..

[7] Klein AP, Lindstrom S, Mendelsohn JB, Steplowski E, Arslan AA, Bas Bueno-de-Mesquita H (2013). An absolute risk model to identify individuals at elevated risk for pancreatic cancer in the general population. PLoS ONE.

[8] Risch HA, Yu H, Lingeng Lu, Kidd MS (2015). Detectable symptomatology preceding the diagnosis of pancreatic cancer and absolute risk of pancreatic cancer diagnosis. Am. J. Epidemiol..

[9] Hippisley-Cox J, Coupland C (2015). Development and validation of risk prediction algorithms to estimate future risk of common cancers in men and women: prospective cohort study. BMJ Open.

[10] Pang, T., Ding, G., Wu, Z., Jiang, G., Yang, Y., Zhang, X. et al. A novel scoring system to analyse combined effect of lifestyle factors on pancreatic cancer risk: a retrospective case-control study. *Sci. Rep.* **7**, 13657 (2017). 10.1038/s41598-017-13182-w PMC5651911 29057932

[11] Kim, J., Yuan, C., Babic, A., Bao, Y., Brais, L. K. & Welch, M. W. Abstract 4945: Absolute risk prediction models for pancreatic cancer. *Cancer Res.* **78**, 4945 (2018).

[12] Nakatochi M, Lin Y, Ito H, Hara K, Kinoshita F, Kobayashi Y (2018). Prediction model for pancreatic cancer risk in the general Japanese population. PLoS ONE.

[13] Wang W, Chen S, Brune KA, Hruban RH, Parmigiani G, Klein AP (2007). PancPRO: risk assessment for individuals with a family history of pancreatic cancer. J. Clin. Oncol..

[14] Cai QC, Chen Y, Xiao Y, Zhu W, Xu QF, Zhong L (2011). A prediction rule for estimating pancreatic cancer risk in chronic pancreatitis patients with focal pancreatic mass lesions with prior negative EUS-FNA cytology. Scand. J. Gastroenterol..

[15] Ruckert F, Brussig T, Kuhn M, Kersting S, Bunk A, Hunger M (2013). Malignancy in chronic pancreatitis: analysis of diagnostic procedures and proposal of a clinical algorithm. Pancreatology.

[16] Boursi B, Finkelman B, Giantonio BJ, Haynes K, Rustgi AK, Rhim AD (2017). A clinical prediction model to assess risk for pancreatic cancer among patients with new-onset diabetes. Gastroenterology.

[17] Sharma A, Kandlakunta H, Singh Nagpal SJ, Feng Z, Hoos W, Petersen GM (2018). Model to determine risk of pancreatic cancer in patients with new-onset diabetes. Gastroenterology.

[18] Radon TP, Massat NJ, Jones R, Alrawashdeh W, Dumartin L, Ennis D (2015). Identification of a three-biomarker panel in urine for early detection of pancreatic adenocarcinoma. Clin. Cancer Res..

[19] Bishop, C. M. *Pattern Recognition and Machine Learning* (Springer, 2006).

[20] Manaswini P, Sahu RK (2011). Multilayer perceptron network in HIV/AIDS application. Int. J. Comput. Appl. Eng. Sci..

[21] Yan H, Jiang Y, Zheng J, Peng C, Li Q (2006). A multilayer perceptron-based medical decision support system for heart disease diagnosis. Expert Syst. Appl..

[22] Shaikhina T, Khovanova NA (2017). Handling limited datasets with neural networks in medical applidations: a small-data approach. Artif. Intell. Med..

[23] Hothorn T, Hornik K, Zeileis A (2006). Unbiased recursive partitioning: a conditional inference framework. J. Comput. Graphical Stat..

[24] Strobl C, Boulesteix AL, Zeileis A, Hothorn T (2007). Bias in random forest variable importance measures: illustrations, sources and a solution. BMC Bioinformatics.

[25] Strobl, C., Boulesteix, A. L., Kneib, T., Augustin, T. & Zeileis, A. Conditional variable importance for random forests. *BMC Bioinformatics* **9**, 307 (2008). 10.1186/1471-2105-9-307 PMC2491635 18620558

[26] Marjanovic, M., Bajat, B. & Kovacevic, M. Landslide susceptibility assessment with machine learning algorithms. In *Proc. International Conference on Intelligent Networking and Collaborative Systems* 273–278 (IEEE, 2009).

[27] Kiseleva EM, Koriashkina LS (2015). Theory of continuous optimal set partitioning problems as a universal mathematical formalism for constructing voronoi diagrams and their generalizations. I. Theoretical foundations. Cybern. Syst. Anal..

[28] Blyuss, O., Koriashkina, L., Kiseleva, E. & Molchanov, R. Optimal placement of irradiation sources in the planning of radiotherapy: mathematical models and methods of solving. *Comput. Math. Methods Med*. **2015**, 142987 (2015). 10.1155/2015/142987 PMC4620430 26543492

[29] Paiva RP, Dourado A (2004). Interpretability and learning in neuro-fuzzy systems. Fuzzy Sets Syst..

[30] Kiseleva EM, Prytomanova OM, Zhuravel SV (2018). Algorithm for solving a continuous problem of optimal partitioning with neurolinguistic identification of functions in target functional. J. Automation Inf. Sci..

[31] Kiseleva EM, Prytomanova OM, Zhuravel SV (2016). Valuation of startups investment attractiveness based on neuro-fuzzy technologies. J. Automation Inf. Sci..

[32] Steyerberg EW, Harrell FE, Borsboom GJ, Eijkemans MJ, Vergouwe Y, Habbema JD (2001). Internal validation of predictive models: efficiency of some procedures for logistic regression analysis. J. Clin. Epidemiol..

[33] Zou H, Hastie T (2005). Regularization and variable selection via the elastic net. J. R. Stat. Soc. B.

[34] Chollet F. *Deep Learning with Python* (Manning Publications Company, 2017).

[35] Pradhan B (2013). A comparative study on the predictive ability of the decision tree, support vector machine and neuro-fuzzy models in landslide susceptibility mapping using GIS. Comput. Geosci..

[36] Kiseleva EM, Koriashkina LS (2015). Theory of continuous optimal set partitioning problems as a universal mathematical formalism for constructing voronoi diagrams and their generalizations. II. Algorithms for constructing Voronoi diagrams based on the theory of optimal set partitioning. Cybern. Syst. Anal..

[37] DeLong ER, DeLong DM, Clarke-Pearson DL (1988). Comparing the areas under two or more correlated receiver operating characteristic curves: a nonparametric approach. Biometrics.

[38] Peduzzi P, Concato J, Kemper E, Holford TR, Feinstein AR (1996). A simulation study of the number of events per variable in logistic regression analysis. J. Clin. Epidemiol..

